# Sulforaphane Decrease of SERTAD1 Expression Triggers G1/S Arrest in Breast Cancer Cells

**DOI:** 10.1089/jmf.2018.4195

**Published:** 2019-05-14

**Authors:** An-Chin Cheng, Ching-Ju Shen, Chao-Ming Hung, Yi-Chiang Hsu

**Affiliations:** ^1^ Department of Nutrition and Health Sciences; College of Health Sciences; Chang Jung Christian University, Tainan, Taiwan.; ^2^ Department of Gynecology and Obstetrics, Kaohsiung Medical University Hospital, Kaohsiung Medical University, Kaohsiung, Taiwan.; ^3^ Department of General Surgery, E-Da Hospital, I-Shou University, Kaohsiung, Taiwan.; ^4^ Department of Medical Sciences Industry, College of Health Sciences; Chang Jung Christian University, Tainan, Taiwan.

**Keywords:** *breast cancer*, *G1/S arrest*, *SERTAD1*, *sulforaphane*

## Abstract

Studies have identified the potential of chemopreventive effects of sulforaphane (SFN); however, the underlying mechanisms of its effect on breast cancer require further elucidation. This study investigated the anticancer effects of SFN that specifically induces G1/S arrest in breast ductal carcinoma (ZR-75-1) cells. The proliferation of the cancer cells after treatment with SFN was detected by 3-(4,5-dimethylthiazol-2-yl)-2,5-diphenyltetrazolium bromide assay. DNA content and cell cycle status were analyzed through flow cytometry. Our results demonstrated the inhibition of growth in ZR-75-1 cells upon SFN exposure. In addition, SERTAD1 (SEI-1) caused the accumulation of SFN-treated G1/S-phase cells. The downregulation of SEI-1, cyclin D2, and histone deacetylase 3 suggested that in addition to the identified effects of SFN against breast cancer prevention, it may also exert antitumor activities in established breast cancer cells. In conclusion, SFN can inhibit growth of and induce cell cycle arrest in cancer cells, suggesting its potential role as an anticancer agent.

## Introduction

Sulforaphane (SFN), a compound within the isothiocyanate group, is a biologically active phytochemical of cruciferous vegetables that has been extensively characterized for its reported anticancer, antimicrobial, and antioxidant properties.^1^ In cooked broccoli and broccoli sprouts, glucoraphanin, a precursor of SFN, requires metabolic conversion to active SFN by myrosinase from gastrointestinal microflora.^2^ After absorption of SFN, it is metabolized into its sequential metabolites, dithiocarbamates.^3^ Some researchers have illustrated several anticancer efficacies of SFN consumption, such as *in vitro* and *in vivo* activities in reducing tumor growth, increasing cancer cell apoptosis, blocking cell cycle progression, and inhibiting signaling within tumor microenvironments.^4–6^

Breast cancer is by far the most prevalent cancer affecting women worldwide. Evidence indicated that dietary consumption of SFN can be distributed to the breast tissue in humans.^7^ Consuming cruciferous vegetables has also been shown to have a function of chemopreventive agent for breast cancer.^8–10^ Several dietary intervention studies indicated that intake of SFN and broccoli was connected with decrements in multiplicity, tumor size, and growth in a rodent model for breast cancer.^7^

Cyclin-dependent kinases (CDKs) are critical regulators controlling progression through the cell cycle.^11^ Disproportion of the cyclin D (CCND) and CDK pathway may cause deregulation of the cell cycle and provoke cancer growth and metastatic potential.^12,13^ In complex with CCND subunits, phosphorylation of the retinoblastoma tumor suppressor protein and CDK4/6 secures DNA replication and thus progression of cells through the cell cycle,^12^ whereas CDK4/6 inhibition has been shown to trigger potent G1 arrest and tumor regression.^11^ SEI-1, a regulatory gene of cell cycle at 19q13.1, is a region frequently amplified in many solid tumors, such as human breast, esophagus, pancreatic, ovarian, and lung cancers.^14,15^ The SEI1 gene product p34SEI1, also known as SERTAD1, is part of the Sertad family,^16^ which is a CDK4-binding protein that works against the inhibiting activity of p16 on cell cycle progression by promoting the association of CDK4–CCND complexes and stimulating CDK4 activity.^17^

This study aims at focusing especially on SFN and its chemopreventive activities against breast cancer cells ZR-75-1. Efforts have been initiated to inspect the effects of SFN on cell growth and cell cycle regulation, and expression levels of downstream molecules were additionally evaluated. The results illustrated that the increase in the G1 population of ZR-75-1 cells can be attributed to the repression of CDK4 after exposure to SFN. The repression of CDK4–CCND complex in ZR-75-1 cells by SFN may be realized through downregulation of SERTAD1gene expression with reducing the CDK4 activity in breast cancer cells.

## Materials and Methods

### Materials

All chemicals and reagents were of analytical grade. SFN, dimethyl sulfoxide (DMSO), and 3-(4,5-dimethylthiazol-2-yl)-2,5-diphenyltetrazolium bromide (MTT) were obtained from Sigma (St Louis, MO, USA). Phosphate-buffered saline, fetal bovine serum (FBS), Dulbecco's modified Eagle medium, sodium pyruvate, trypsin, and antibiotics were obtained from Gibco, BRL (Grand Island, NY, USA). Annexin V-FITC was obtained from BD Pharmingen (USA). Molecular weight markers were obtained from Bio-Rad (USA), and polyvinylidene fluoride (PVDF) membranes were purchased from Millipore.

### Cells

ZR-75-1 (NCI-PBCF-CRL1500) cells, which are breast ductal carcinoma cells, were purchased from Bioresource Collection and Research Center (BCRC, Hsinchu, Taiwan). The complete growth medium used to expand ZR-75-1 cells is RPMI media 1640 supplemented with 10% (v/v) FBS. The ZR-75-1 cells were incubated in a humidified incubator in an atmosphere of 95% air and 5% carbon dioxide at 37°C.

### Cell proliferation assay

The cells were placed into a 96-well plate at 5000 cells per well followed by exposure to 0, 6.25, 12.5, or 25 *μ*M SFN for 1–3 days. MTT (1 mg/mL) solution was added to each well for at least 4 h. The reaction was blocked by adding DMSO, and measured at 540 nm using a multiwell plate reader (*μ*QUANT; BioTek Instruments, Inc., USA). Background absorbance (medium without cells) was subtracted. All samples were assayed at least in triplicate, and the mean was calculated for each experiment.

### Apoptosis measurement

The cells were cultured in six-well culture plates (Orange Scientific, EU). After exposure to SFN for 4 h, the cells were harvested by centrifugation, resuspended in and incubated with 1 × annexin-binding buffer containing 5 *μ*L of annexin V-FITC and 1 *μ*L of propidium iodide (PI) (100 *μ*g/mL), and incubated at room temperature for 15 min. The stained cells were analyzed on an FACSCalibur flow cytometer (BD Pharmingen) using WinMDI 2.9 free software (BD Pharmingen).

### Cell cycle analysis

To facilitate cell cycle analysis, a fluorescent nucleic acid dye PI was used to identify the proportion of cells in each of the three interphase stages. The cells were treated with SFN for 24 h followed by harvesting and fixing in 1 mL of ice-cold ethanol (70%) at −20°C for at least 8 h. DNA was stained with PI/RNaseA staining buffer, and the cell cycle was analyzed using an FACSCalibur flow cytometer. Data were interpreted using WinMDI 2.9 software.

### Western blot analysis

Proteins (50–75 *μ*g) were loaded onto 10–12% sodium dodecyl sulfate-polyacrylamide gel electrophoresis membranes for electrophoretic separation and then transferred to PVDF membranes. After blocking overnight with Odyssey blocking buffer (USA), the membranes were incubated with anti-*β*-actin (Sigma-Aldrich) and anti-CDK2 (H-298: sc-748), anti-CDK4 (H-22: sc-601), anti-CDK6 (C-21: sc-177), anti-CCND (M-20: sc-718), cyclin D2 (CCND2) (M-20: sc-593), SERTAD1 (H-70: sc-135012), and histone deacetylase 3 (HDAC3) (H-99: sc-11417) (Santa Cruz BioTechnology, USA) antibodies for 90–120 min. The membranes were washed several times and then incubated with a corresponding secondary antibody (IRDye Li-COR, USA) at a dilution of 1:20,000 for 30–45 min. Antigens were then visualized using a near-infrared fluorescence imaging system (Odyssey LI-COR, USA), and the data were interpreted using the Odyssey 2.1 software or a chemiluminescence detection kit (ECL; Amersham Corp., Arlington Heights, IL, USA).

### Quantitative real-time PCR

Quantitative real-time PCR (qRT-PCR) was carried out using an ABI 7300 Real-Time PCR system (Applied Biosystems, Foster City, CA, USA) with SYBR-Green under the condition of 40 cycles: 95°C for 120 sec, 60°C for 30 sec, and 72°C for 30 sec. All samples were run in duplicate and the threshold suggested by the software was adopted for Ct calculations. In this study, we used Ct values obtained at 18 sec as internal controls for each run and a delta Ct value for each tested gene. All protocols were carried out following the manufacturer's instructions.

### Statistical analysis

Data are expressed as the mean ± standard error of the mean of at least three independent experiments. Student's *t*-test or one-way analysis of variance with a Scheffe's *post hoc* test was used for statistical analysis. A *P*-value of <.05 was considered as statistically significant.

## Results

### SFN inhibits the survival and proliferation of ZR-75-1 cells

**FIG. 1.**
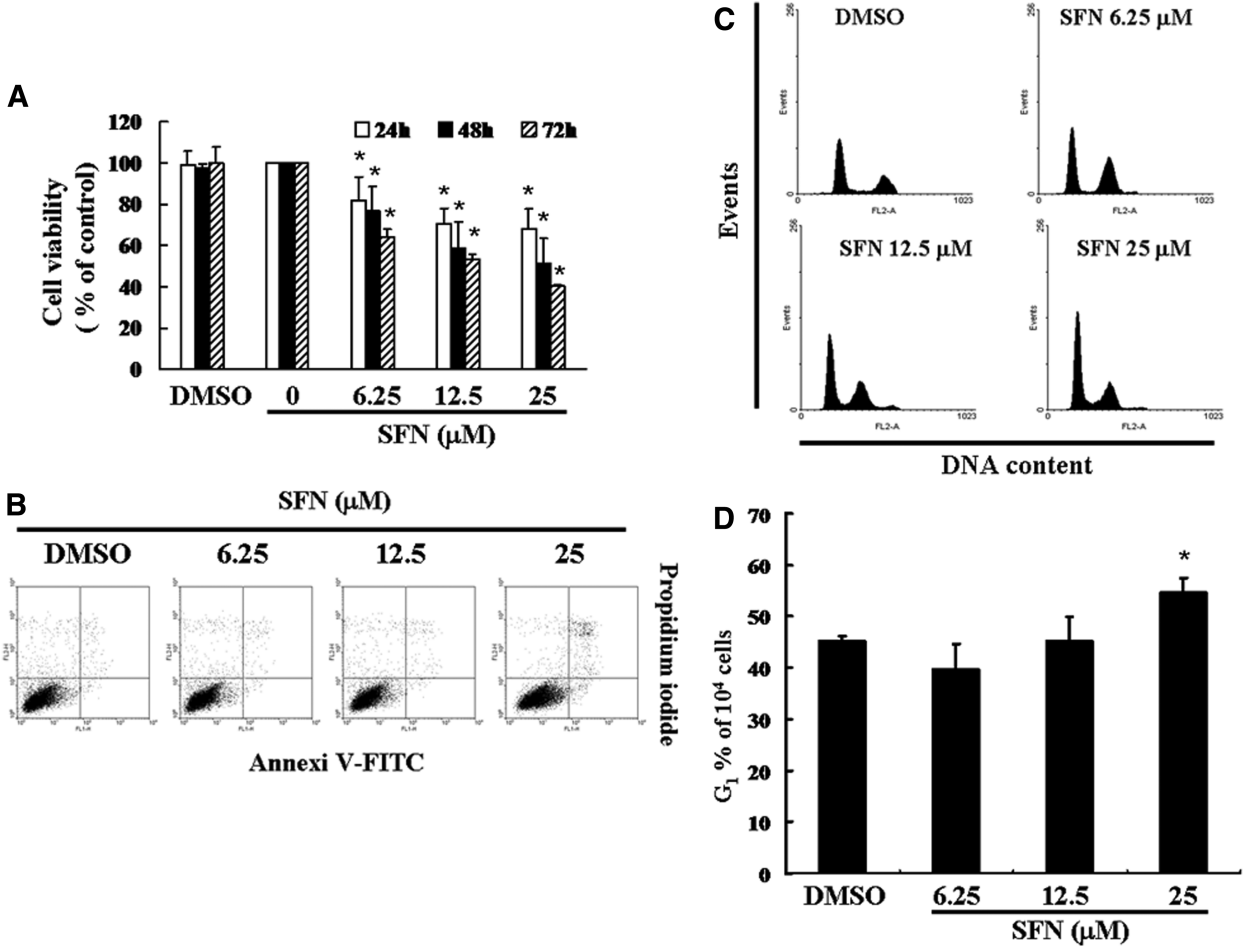
SFN mediates the survival of ZR-75-1 cells by inhibiting proliferation: **(A)** *In vitro* study was initiated by treating ZR-75-1 cells with increasing doses of SFN (0, 6.25, 12.5, and 25 *μ*M) for 1–3 days. The survival of SFN-treated cancer cells was then measured by the MTT method. Results are expressed as a percentage of control, which was considered 100%. All data were reported as the mean (±SEM) of at least three separate experiments. Statistical analysis was performed using a *t*-test, with significant differences determined at the level of *P < .05 versus the control group. **(B)** The effects of SFN on apoptosis/necrosis in ZR-75-1 cells. **(C)** The cell cycle analysis of the cancer cells after being cultured with SFN for 24 h. **(D)** SFN induced an increase in the G1-phase cell percentage (%). Cells underwent staining with propidium iodide to analyze DNA content, which was then quantified through flow cytometry. In each group of bars, * indicates that the number of G1 cells in the SFN treatment group was significantly higher than that of the control group (*P* < .05). DMSO, dimethyl sulfoxide; MTT, 3-(4,5-dimethylthiazol-2-yl)-2,5-diphenyltetrazolium bromide; SEM, standard error of the mean; SFN, sulforaphane.

To inspect the effects of SFN on the cell survival and proliferation, *in vitro* study was applied to treat ZR-75-1 cells with different concentrations of SFN (0, 6.25, 12.5, and 25 *μ*M) for 24–72 h, while cell viability was detected by the MTT assay. As shown in Figure 1A, the survival ratios of ZR-75-1 cells were significantly reduced by SFN in a time- and dose-dependent pattern.

### Non-SFN-induced apoptosis/necrosis of ZR-75-1 cells

To clarify the role of SFN in the apoptosis/necrosis of breast cancer cells, the cells were treated with SFN for 4 h followed by detecting the generation of sub-G1 cells by Annexin V-FITC and PI staining. Sub-G1 cell population and apoptotic ratios were analyzed by flow cytometry. Compared with the untreated (control) cells, an Annexin-FITC/PI assay exhibited nonsignificant changes in the percentage of apoptosis or necrosis on SFN-treated cells (Fig. 1B). Moreover, no significant rise in the percentage of caspase-3 activity was detected in SFN-treated cancer cells (data not shown). Therefore, these results indicated that incubation with SFN inhibited cell survival and proliferation, but did not undergo cell apoptosis or necrosis.

### SFN-induced accumulation of G1 phase in ZR-75-1 cells

To further investigate the effect of SFN on ZR-75-1 cell growth, the cell cycle distribution among SFN-treated cells was analyzed and quantified by flow cytometry. Cells were treated with SFN for 24 h followed by processing and analysis. As shown in Figure 1C and D, treatment with SFN led to an increment in the G1 phase cell population, implying that ZR-75-1 cells underwent a delay in G1/S phase checkpoint. These results suggested that exposure to SFN enhanced cell populations in the G1/S phase while synchronously decreasing the S phase population (data not shown).

### SFN-induced cell cycle arrest in ZR-75-1 cells through downregulation of CDK4

**FIG. 2.**
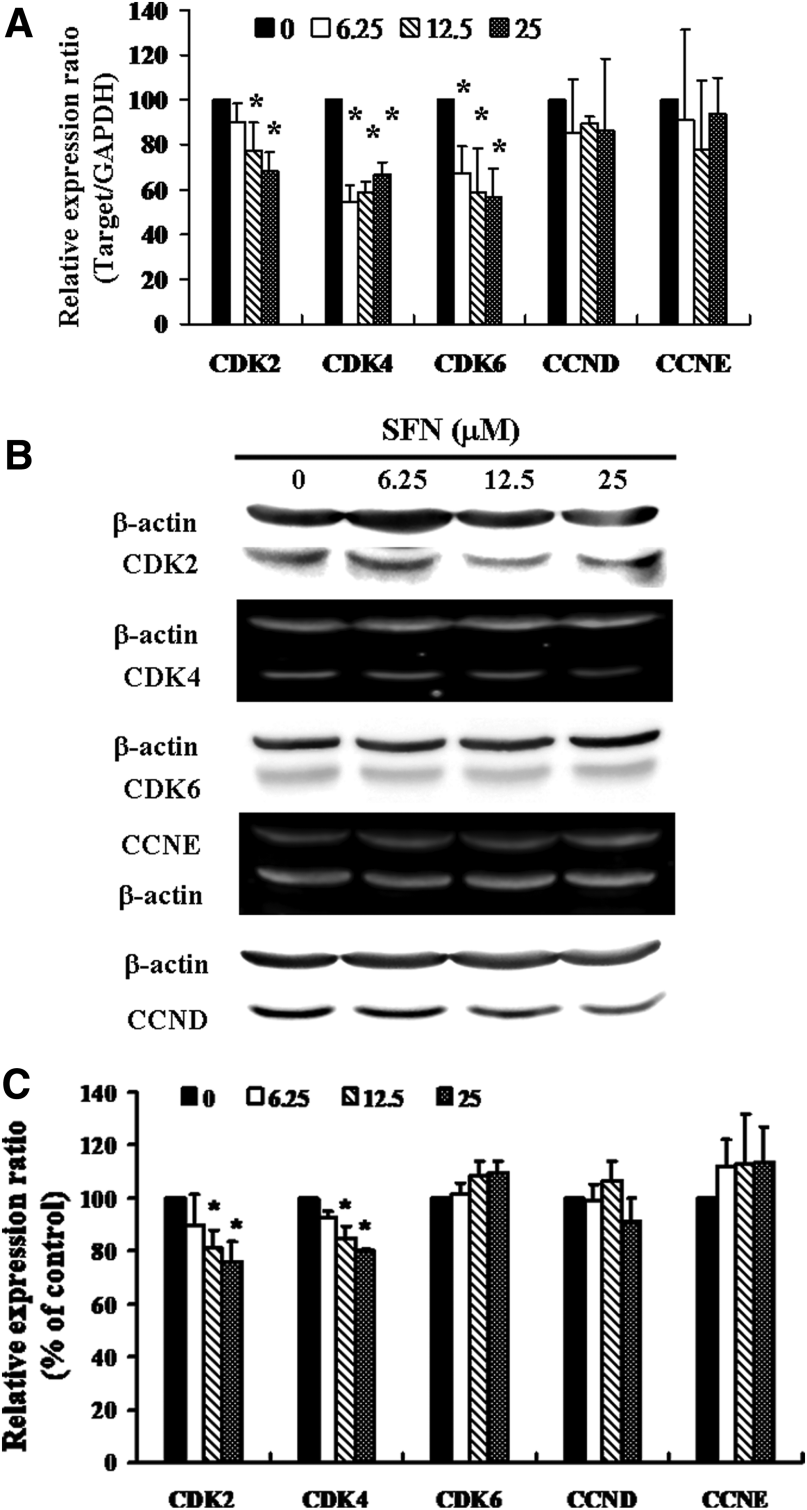
SFN represses expression of CDKs in ZR-75-1 cells. Cells were treated with SFN for 24 h, the gene and protein expression was subsequently detected using **(A)** qRT-PCR and **(B)** Western blot analysis. **(C)** Representative blots from three independent experiments. Quantification of band intensities. All data are reported as the mean (±SEM) of at least three separate experiments. Statistical analysis was performed using the *t*-test, with differences considered significant at a level of *P < .05 versus the 0 *μ*M SFN control group. CDKs, cyclin-dependent kinases; GAPDH, glyceraldehyde-3-phosphate dehydrogenase; qRT-PCR, quantitative real-time PCR.

Figure 2A presents the results of qRT-PCR of SFN-treated ZR-75-1 cells. In this test, we measure relative intensities to quantify the gene expression levels of CDKs, CCND, and cyclin E (CCNE). The results revealed that the messenger RNA (mRNA) levels of CDK2, CDK4, and CDK6 were reduced after incubation with SFN compared with that of control group (Fig. 2A). There were no significant variations in the mRNA expression levels of CCND and CCNE. To further clarify the effect of SFN on cell cycle-related proteins in ZR-75-1 cells, Western blot analysis was used. Figure 2B and C shows that there were no significant differences in the expression of CDK6, CCND, and CCNE proteins. However, the protein levels of CDK2 and CDK4 were significantly downregulated in those cells exposed to SFN at concentrations of 12.5 and 25 *μ*M (Fig. 2B, C).

### Effects of SFN on SERTAD1 in ZR-75-1 cells

**FIG. 3.**
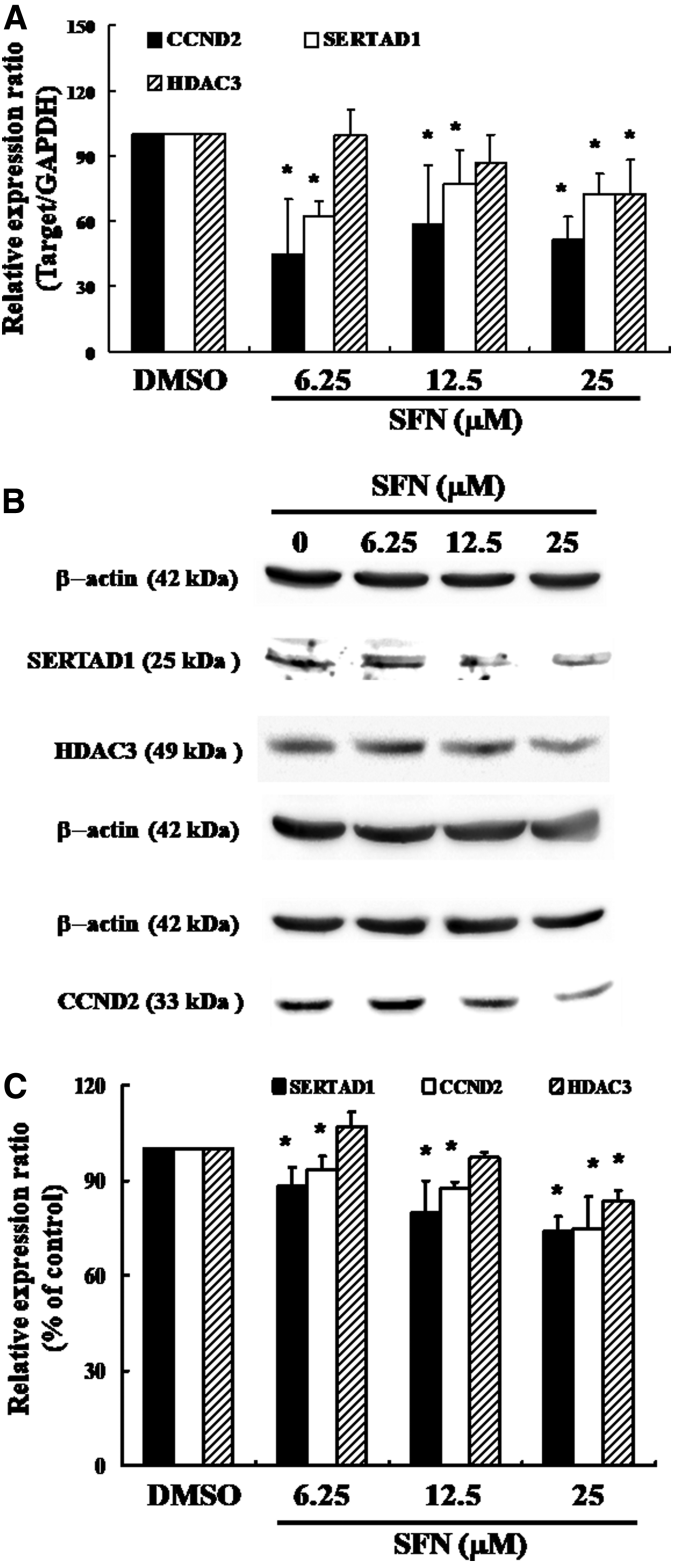
SERTAD1 and HDAC3 gene expression in ZR-75-1 cells after exposure to SFN: **(A)** qPCR and **(B)** Western blotting analysis of SERTAD1, CCND2, and HDAC3 gene expression standardized against the levels of *β*-actin in cancer cell lines exposed to DMSO (SFN 0 *μ*M control) or SFN group. **(C)** Representative blots from three independent experiments. Quantification of band intensities. All data were reported as the mean (±SEM) of at least three separate experiments. Statistical analysis was performed using a *t*-test, with differences considered significant at a level of *P < .05 versus the 0 *μ*M SFN control group. CCND2, cyclin D2; DMSO, dimethyl sulfoxide; HDAC3, histone deacetylase 3.

Figure 3A shows the results of qRT-PCR of SFN-treated ZR-75-1 cells. In this test, SERTAD1, CCND2, and HDAC3 gene expression levels were quantified by measuring relative band intensities. The results demonstrated that the mRNA levels of SERTAD1, CCND2, and HDAC3 were decreased significantly after incubation with 25 *μ*M SFN (Fig. 3A). Figure 3B shows the results of Western blotting analysis for cellular proteins SERTAD1, CCND2, and HDAC3 expression in SFN-treated breast cancer cells. Each protein was quantified by measuring relative band intensities (Fig. 3C). Western blot analysis showed a noticeable decrease in the SERTAD1and CCDN2 in a dose-dependent pattern after incubation with SFN in ZR-75-1 cells; furthermore, HDAC3 expression was decreased in 25 *μ*M SFN treatment (Fig. 3B, C).

## Discussion

Consuming SFN has been shown to have a protective role for breast cancer, and SFN metabolites were perceptible immediately in human breast tissue after oral dosing.^7^ This study hypothesized that SFN can inhibit the survival of ZR-75-1 breast cancer cells and thereby prevent their proliferation. The MTT assay indicated that the survival of the ZR-75-1 cells was significantly inhibited by SFN in a time- and dose-dependent manner (Fig. 1A). However, as shown in Figure 1B, SFN did not induce cell apoptosis or necrosis in ZR-75-1 cells. Thus, the distribution of cell cycle among SFN-treated cells was further investigated through flow cytometry. The results have demonstrated that treatment with SFN triggers G1/S cell cycle arrest in ZR-75-1 cells (Fig. 1C, D).

The G1/S transition is a step in the cell cycle at the boundary between the G1 phase and the S phase.^18^ Previous studies have indicated that the CDK-RB-E2F pathway plays a relevant role for cell cycle progression. During this transition, the G1/S specific CDK activities are composed of complexes between CCND and CDK4/6, as well as between CCNE and CDK2 in mammalian cells.^19^ The cylcin–CDK targets retinoblastoma (Rb) protein for phosphorylation. Upon phosphorylation, transcription factor E2F is released and activated, allowing progression of G1 to S phase.^20,21^ Many checkpoints are deregulated in cancer cells, and this is often as a result of alterations of the cell cycle regulatory machinery and cyclin–CDK complexes.^22^ As shown in Figure 2C, the protein expression of CDK2 was decreased significantly in SFN-treated ZR-75-1 cells. We proposed that the late G1 CCNE–CDK2 complex may be involved in the SFN-induced G1-S cell cycle arrest. We have also checked the early and mid G1 stage-specific complex between CCND and CDK4/6, finding that there were no marked variations in the protein level of CCND and CDK6. Interestingly to point, compared with the 6.5 *μ*m treatment, Western blot analysis showed that CDK4 was downregulated essentially in SFN-treated cells at concentration of 12.5 and 25 *μ*M (Fig. 2B, C), whereas the mRNA level of CDK4 was increased (Fig. 2A). Therefore, we presume that the opposite exhibitions of the mRNA and protein of CDK4 may be due to post-transcriptional modification; in the meantime, an increase in the number of ZR-75-1 cells in the G1 phase can be based on the repression of CDK4 after incubation with SFN.

Previously, our laboratory demonstrated that SFN could induce cell cycle G1/S delay in MDA-MB-231 breast cancer cells, and outcomes from qPCR analysis were further approved using microarray analysis, indicating substantial SERTAD1, CCDN2, and HDAC3 downregulation, as well as the noticeable repression of CDK4 after exposure to SFN. Thus, we have further investigated the mRNA and protein levels of HDAC3, CCDN2, and SERTAD1 in SFN-treated ZR-75-1 cells. HDAC activity links the DNA damage and repair pathways.^23^ SFN could suppress HDAC activity and result in histone hyperacetylation in cancer cells.^24^ Among the class I HDACs, HDAC3 responded as the earliest one and was the most sensitive to SFN-suppressed protein expression.^25^ Therefore, we evaluated HDAC3 expression to verify its effect on the SFN-induced cell cycle arrest. Our results indicated that protein expression of HDAC3 was decreased markedly in 25 *μ*M SFN treatment (Fig. 3B, C), and it may be responsible for the SFN-triggered G1/S arrest in ZR-75-1 cells at 25 *μ*M treatment.

The oncogene, CCND2, is overexpressed in breast cancer,^26^ and it is characterized as the regulator of G1 to S-phase transition during the cell cycle.^27,28^ The critical function of CCND2 is attributed to assemble a complex with CDK4/CDK6, motivate the phosphorylation of Rb protein, releasing the E2F transcription factor, and, subsequently, activating the genes transcription involved in the progression of G1 to S phase.^26,27^ As shown in Figure 3, compared with the control groups, there was a noticeable decrement in the mRNA and protein expression of CCND2 in SFN-treated cells. Our study has demonstrated that CCND2 is involved in SFN-triggered G1/S arrest in ZR-75-1 cells.

SERTAD1 was suggested as a positive regulator of the cell cycle,^15,17,29,30^ and the potential oncogenic effects of SERTAD1 are suggested by its expression that is upregulated in several types of tumors.^29,31–33^ SERTAD1 can bind to CDK4 directly and form a quaternary complex with CCND2 and p16 to mediate CDK4 activity.^34^ Several studies indicated that overexpression of SERTAD1 can provoke hyperproliferation^17,30^ and inhibition of apoptosis.^35^ However, a direct link between SERTAD1and cancer pathogenesis remains obscure. Therefore, our discussion will focus mainly on the role of SERTAD1 on SFN-treated ZR-75-1 cells. The results revealed a dramatic reduction in the mRNA and protein levels of SERTAD1 after incubation with SFN in ZR-75-1 cells (Fig. 3). As shown in Figure 2B and C, we suggested that SFN can trigger G1/S arrest inZR-75-1 cells through repression of CDK4. Accordingly, the expression of both SERTAD1 and CCND2 also decreased significantly after incubation with SFN in ZR-75-1 cells. Given this overall information, we propose a novel pathway in which SFN could defer cancer cell growth in the G1 phase through downregulation of SERTAD1 proteins followed by dissociation of the CCND–CDK4 complex.

## References

[1] Zhang Y, Tang L: Discovery and development of sulforaphane as a cancer chemopreventive phytochemical. Acta Pharmacol Sin 2007;28:1343–1354 17723168 10.1111/j.1745-7254.2007.00679.x

[2] Song L, Thornalley PJ: Effect of storage, processing and cooking on glucosinolate content of Brassica vegetables. Food Chem Toxico 2007;45:216–224 10.1016/j.fct.2006.07.021 17011103

[3] Amjad AI, Parikh RA, Appleman LJ, Hahm ER, Singh K, Singh SV: Broccoli-derived sulforaphane and chemoprevention of prostate cancer: From bench to bedside. Curr Pharmacol Rep 2015;1:382–390 26557472 10.1007/s40495-015-0034-x PMC4635516

[4] Atwell LL, Beaver LM, Shannon J, Williams DE, Dashwood RH, Ho E: Epigenetic regulation by sulforaphane: Opportunities for breast and prostate cancer chemoprevention. Curr Pharmacol Rep 2015;1:102–111 26042194 10.1007/s40495-014-0002-x PMC4450146

[5] Singh SV, Herman-Antosiewicz A, Singh AV, *et al.*: Sulforaphane-induced G2/M phase cell cycle arrest involves checkpoint kinase 2-mediated phosphorylation of cell division cycle 25C. J Biol Chem 2004;279:25813–25822 15073169 10.1074/jbc.M313538200

[6] Clarke JD, Hsu A, Yu Z, Dashwood RH, Ho E: Differential effects of sulforaphane on histone deacetylases, cell cycle arrest and apoptosis in normal prostate cells versus hyperplastic and cancerous prostate cells. Mol Nutr Food Res 2011;55:999–1009 21374800 10.1002/mnfr.201000547 PMC3129466

[7] Cornblatt BS, Ye L, Dinkova-Kostova AT, *et al.*: Preclinical and clinical evaluation of sulforaphane for chemoprevention in the breast. Carcinogenesis 2007;28:1485–1490 17347138 10.1093/carcin/bgm049

[8] Terry P, Wolk A, Persson I, Magnusson C: Brassica vegetables and breast cancer risk. JAMA 2001;285:2975–2977 11410091 10.1001/jama.285.23.2975

[9] Fowke JH, Chung FL, Jin F, *et al.*: Urinary isothiocyanate levels, brassica, and human breast cancer. Cancer Res 2003;63:3980–3986 12873994

[10] Ambrosone CB, McCann SE, Freudenheim JL, Marshall JR, Zhang Y, Shields PG: Breast cancer risk in premenopausal women is inversely associated with consumption of broccoli, a source of isothiocyanates, but is not modified by GST genotype. J Nutr 2004;134:1134–1138 15113959 10.1093/jn/134.5.1134

[11] Shapiro GI: Cyclin-dependent kinase pathways as targets for cancer treatment. J Clin Oncol 2006;24:1770–1783 16603719 10.1200/JCO.2005.03.7689

[12] Murphy CG, Dickler MN: The Role of CDK4/6 Inhibition in Breast Cancer. Oncologist 2015;20:483–490 25876993 10.1634/theoncologist.2014-0443 PMC4425391

[13] Mayer EL: Targeting breast cancer with CDK inhibitors. Curr Oncol Rep 2015;17:443 25716100 10.1007/s11912-015-0443-3

[14] Gronwald J, Jauch A, Cybulski C, *et al.*: Comparison of genomic abnormalities between BRCAX and sporadic breast cancers studied by comparative genomic hybridization. Int J Cancer 2005;114:230–236 15540206 10.1002/ijc.20723

[15] Hsu SI, Yang CM, Sim KG, Hentschel DM, O'Leary E, Bonventre JV: TRIP-Br: A novel family of PHD zinc finger- and bromodomain-interacting proteins that regulate the transcriptional activity of E2F-1/DP-1. EMBO J 2001;20:2273–2285 11331592 10.1093/emboj/20.9.2273 PMC125435

[16] Lai IL, Wang SY, Yao YL, Yang WM: Transcriptional and subcellular regulation of the TRIP-Br family. Gene 2007;388:102–109 17141982 10.1016/j.gene.2006.10.008

[17] Sugimoto M, Nakamura T, Ohtani N, *et al.*: Regulation of CDK4 activity by a novel CDK4-binding protein, p34(SEI-1). Genes Dev 1999;13:3027–3033 10580009 10.1101/gad.13.22.3027 PMC317153

[18] Bartek J, Lukas J: Pathways governing G1/S transition and their response to DNA damage. FEBS Lett 2001;490:117–122 11223026 10.1016/s0014-5793(01)02114-7

[19] Reed SI: Control of the G1/S transition. Cancer Surv 1997;29:7–23 9338094

[20] Johnson J, Thijssen B, McDermott U, Garnett M, Wessels LF, Bernards R: Targeting the RB-E2F pathway in breast cancer. Oncogene 2016;35:4829–4835 26923330 10.1038/onc.2016.32 PMC4950965

[21] Thwaites MJ, Cecchini MJ, Passos DT, Welch I, Dick FA: Interchangeable roles for E2F transcriptional repression by the retinoblastoma protein and p27KIP1-CDK regulation in cell cycle control and tumor suppression. Mol Cell Biol 2017;4:37 10.1128/MCB.00561-16 PMC5214858 27821477

[22] Ravitz MJ, Wenner CE: Cyclin-dependent kinase regulation during G1 phase and cell cycle regulation by TGF-beta. Adv Cancer Res 1997;71:165–207 9111866 10.1016/s0065-230x(08)60099-8

[23] Robert T, Vanoli F, Chiolo I, *et al.*: HDACs link the DNA damage response, processing of double-strand breaks and autophagy. Nature 2011;471:74–79 21368826 10.1038/nature09803 PMC3935290

[24] Myzak MC, Karplus PA, Chung FL, Dashwood RH: A novel mechanism of chemoprotection by sulforaphane: Inhibition of histone deacetylase. Cancer Res 2004;64:5767–5774 15313918 10.1158/0008-5472.CAN-04-1326

[25] Rajendran P, Delage B, Dashwood WM, *et al.*: Histone deacetylase turnover and recovery in sulforaphane-treated colon cancer cells: Competing actions of 14-3-3 and Pin1 in HDAC3/SMRT corepressor complex dissociation/reassembly. Mol Cancer 2011;10:68 21624135 10.1186/1476-4598-10-68 PMC3127849

[26] Zhang P: The cell cycle and development: Redundant roles of cell cycle regulators. Curr Opin Cell Biol 1999;11:655–662 10600701 10.1016/s0955-0674(99)00032-0

[27] Evron E, Umbricht CB, Korz D, *et al.*: Loss of cyclin D2 expression in the majority of breast cancers is associated with promoter hypermethylation. Cancer Res 2001;61:2782–2787 11289162

[28] Yu J, Leung WK, Ebert MP, *et al.*: Absence of cyclin D2 expression is associated with promoter hypermethylation in gastric cancer. Br J Cancer 2003;88:1560–1565 12771922 10.1038/sj.bjc.6600940 PMC2377112

[29] Li J, Muscarella P, Joo SH, *et al.*: Dissection of CDK4-binding and transactivation activities of p34(SEI-1) and comparison between functions of p34(SEI-1) and p16(INK4A). Biochemistry 2005;44:13246–13256 16201750 10.1021/bi0504658

[30] Tang DJ, Hu L, Xie D, *et al.*: Oncogenic transformation by SEI-1 is associated with chromosomal instability. Cancer Res 2005;65:6504–6508 16061626 10.1158/0008-5472.CAN-05-0351

[31] Fernandez-Marcos PJ, Pantoja C, Gonzalez-Rodriguez A, *et al.*: Normal proliferation and tumorigenesis but impaired pancreatic function in mice lacking the cell cycle regulator sei1. PLoS One 2010;5:e8744 20090907 10.1371/journal.pone.0008744 PMC2807453

[32] Tang TC, Sham JS, Xie D, *et al.*: Identification of a candidate oncogene SEI-1 within a minimal amplified region at 19q13.1 in ovarian cancer cell lines. Cancer Res 2002;62:7157–7161 12499249

[33] van Dekken H, Alers JC, Riegman PH, Rosenberg C, Tilanus HW, Vissers K: Molecular cytogenetic evaluation of gastric cardia adenocarcinoma and precursor lesions. Am J Pathol 2001;158:1961–1967 11395372 10.1016/S0002-9440(10)64666-4 PMC1891976

[34] Li J, Melvin WS, Tsai MD, Muscarella P: The nuclear protein p34SEI-1 regulates the kinase activity of cyclin-dependent kinase 4 in a concentration-dependent manner. Biochemistry 2004;43:4394–4399 15065884 10.1021/bi035601s

[35] Hong SW, Kim CJ, Park WS, *et al.*: p34SEI-1 inhibits apoptosis through the stabilization of the X-linked inhibitor of apoptosis protein: p34SEI-1 as a novel target for anti-breast cancer strategies. Cancer Res 2009;69:741–746 19176394 10.1158/0008-5472.CAN-08-1189

